# Neural network ensemble model for prediction of erythrocyte sedimentation rate (ESR) using partial least squares regression

**DOI:** 10.1038/s41598-022-23174-0

**Published:** 2022-11-15

**Authors:** Jaejin Lee, Hyeonji Hong, Jae Min Song, Eunseop Yeom

**Affiliations:** 1 School of Mechanical Engineering, Pusan National University, Busan, South Korea; 2 Department of Oral and Maxillofacial Surgery, School of Dentistry, Pusan National University, Yangsan, South Korea; 3 Dental and Life Science Institute, School of Dentistry, Pusan National University, Yangsan, South Korea

**Keywords:** Biomedical engineering, Mechanical engineering, Biomarkers

## Abstract

The erythrocyte sedimentation rate (ESR) is a non-specific blood test for determining inflammatory conditions. However, the long measurement time (60 min) to obtain ESR is an obstacle for a prompt evaluation. In this study, to reduce the measurement time of ESR, deep neural networks (DNNs) were applied to the sedimentation tendency of blood samples. DNNs using multilayer perceptron (MLP), long short-term memory (LSTM), and gated recurrent unit (GRU) were assessed and compared to determine a suitable length of time for the input sequence. To avoid overfitting, a stacking ensemble learning was adopted, which combines multiple models by using a meta model. Four meta models were compared: mean, median, least absolute shrinkage and selection operator, and partial least squares regression (PLSR) schemes. From the empirical results, LSTM and GRU models have better prediction than MLP over sequence lengths of 5 to 20 min. The decrease in $\overline{\mathrm{MAPE} }$ and $\overline{\mathrm{RMSE} }$ of GRU and LSTM was attenuated after a sequence length of 15 min, so the input sequence length is determined as 15 min. In terms of the meta model, the statistical comparison suggests that GRU combined with PLSR (GRU–PLSR) is the best case. Then, the GRU–PLSR was tested for prediction of ESR data obtained from periodontitis patients to check its applicability to a specific disease. The Bland–Altman plot shows acceptable agreement between measured and predicted ESR values. Based on the results, the GRU–PLSR can predict ESR with improved performance within 15 min and has potential applicability to ESR data with inflammatory and non-inflammatory conditions.

## Introduction

The erythrocyte sedimentation rate (ESR) is a simple and inexpensive blood test for providing general information about an inflammatory or acute response. As the gold standard of ESR measurements, the Westergren method measures the distance from the meniscus to the top of the column of sedimented erythrocytes separated from plasma in a vertical Westergren tube (inner diameter = 2.5 mm, length = 200 mm, and whole blood volume = 5 mL) after 1 h^1,2^. The ESR value represents the sedimentation speed in units of millimeters per hour.

Under low shear conditions, erythrocytes tend to form one-dimensional coin stacks (rouleaux) or three-dimensional aggregates in a reversible process. Erythrocyte aggregation (EA) is affected by two major factors: aggregating force induced by the macromolecules, and disaggregating forces including negative surface charge and shear force^3^. Although negative charges of erythrocytes repel each other, the presence of certain plasma proteins (fibrinogen, immunoglobulins, lipoproteins, and α-2 macroglobulin) and inflammation-related proteins (cytokines and chemokines) in plasma enhance the degree of EA. Elevated EA is considered to augment blood viscosity^4^, which increases flow resistance and wall shear stress. In addition, the high extent of EA is somewhat correlated with cardiovascular diseases (CVDs)^4^ and diabetes mellitus^5^. Considering Stokes’ law, the sedimentation speed of a spherical particle in a medium is dependent on its size^6^. Therefore, the ESR value is closely associated with the degree of EA^7^.

Although ESR with minor modifications is still considered to be a useful index of nonspecific disease, the Westergren method has some disadvantages, including a time-consuming procedure (60 min) and a repetitive cleaning process. In addition, the Westergren method provides only the final sedimentation speed (the ESR value) after 1 h. It is insufficient to describe the temporal variation of the sedimentation tendency. Hong et al. proposed the micro-vibrational erythrocyte sedimentation rate (MV-ESR) and demonstrated the possibility of shortening the time of ESR measurement^8^. Shteinshnaider et al. demonstrated that ESR values at 60 min can be predicted by ESR values at 30 min by using simple linear regression^9^. Nishisako et al. applied a formula for calculating a normal maximum range of ESR with only age information of patients^10^. However, this formula was only applicable to Asian patients aged more than 65 years. Lapić et al. compared automated ESR methods (TEST1, Ves-Matic Cube 200) with the Westergren method^11^. Ves-Matic Cube 200 showed larger dispersion, so they concluded that automated ESR methods should be used carefully. To gain information about the temporal sedimentation tendency, a characteristic time representing the time required for sedimentation by a specific length was proposed^12^.

Although ESR elevation typically results from inflammatory factor such as fibrinogen, some noninflammatory factors such as age, sex, and pregnancy can also cause elevation of the ESR, which might make it difficult to identify the patterns of ESR^13^. For analysis of complex biological systems, machine learning algorithms have been proposed to recognize, classify, and predict the patterns^14^. Thus, deep learning methods were used in this study considering their ability to learn the various patterns in ESR.

The sedimentation tendency of erythrocytes is time series data. For forecasting a time series, several deep neural networks (DNNs) have been proposed in several fields^15–19^. Multilayer perceptron (MLP) is a type of feed-forward neural network that does not have cycles in the same layer. Unlike the MLP, long short-term memory (LSTM) and gated recurrent unit (GRU) have cycles within a hidden layer and filter the information through gate structure^15,16^. Kong et al. compared various benchmarks with LSTM, including the k-Nearest Neighbors’ algorithm and extreme learning machine^17^. They found that LSTM outperformed the other rival algorithms in short-term forecasting for loads in individual residential households. AlDahoul et al. investigated the usage of ElasticNet linear regression, MLP, extreme gradient boosting, and LSTM for the prediction of suspended sediment in the Johor river in Malaysia^18^. The results showed that the LSTM is better than the other models. Che et al. developed a GRU-based model (GRU-D) that captures the informative missing patterns in time series analysis^19^. It was shown that GRU-D performed better than deep learning models built on GRU as well as other machine learning methods. Yilmaz and Buyrukoğlu proposed the hybrid model combining Back Propagation-Based Artificial Neural Network (BP-ANN), Correlated Additive Model (CAM) and Auto-Regressive Integrated Moving Average (ARIMA) to forecast Coronavirus disease (Covid-19) case^20^. They showed that the proposed hybrid model provides the best results, which can be used as the prediction model for Covid-19 to obtain better prediction results and to take immediate measures. Buyrukoğlu analyzed five promising cryptocurrencies with the ensembles of LSTM and single-based LSTM networks^21^. This study revealed both ensembles of LSTM and single-based LSTM can be used separately, considering that ensemble of LSTM does not always outperform the single-based LSTM.

When a small amount of data is available, a single neural network algorithm tends to exhibit generalization error for unseen instances^22^. Generalization means the capacity of the model to predict the correct output for previously unseen data^23^. Because small datasets are more prone to overfitting than large datasets, an ensemble approach was proposed to reduce the generalization error by avoiding overfitting^24^. Specifically, ensemble learning refers to the combination of multiple algorithms with different opinions and then weighting and combining them to make a complex decision. Ensemble learning is mainly used to improve prediction performance, or to avoid selection of a poor predictions by combining multiple models. In several studies, ensemble learning could improve the forecasting performance^25–27^.

There are commonly used ensemble methods like bagging, boosting, and stacking. Bagging, also known as bootstrap aggregation, constructs multiple classifiers trained on bootstrap samples and averages the outputs of the predictors to obtain the final prediction with the appropriate size^28^. Since the bootstrap samples are randomly drawn with replacement from the entire training data, it causes individual bootstrap samples to overlap significantly, with many of the same instances in the training data appearing in most samples, and some instances appearing multiple times in a given sample. Unlike bagging, boosting creates different classifiers by sequentially reweighting the instances in the training data^29^. Each instance misclassified by the previous base classifier get larger weights, and the next classifier is boosted on the reweighted training data. In this way, a sequence of training data and classifiers is obtained, which can be combined by a simple majority voting or by weight majority voting in the final decision. In stacking, which stands for stacked generalization, a set of base models are constructed from the training data, then their outputs are used to train a meta model^30^. In most cases, the performance of an ensemble is better than a single model^21,31–34^. Ensemble learning has been widely used in various fields such as: health science^31^, sport science^32^, agriculture^33,34^, finance^21^.

In this study, the MLP, LSTM, and GRU models were compared to reduce the measurement time of ESR. Specifically, the sequence length (input data) is determined by monitoring predictive performance with evaluation metrics for each model. After determining the sequence length, stacking ensemble models with different ensemble sizes were investigated to select an adequate ensemble structure. Furthermore, all models including the individual DNNs were compared statistically. From the statistical comparison, the best performing meta model was determined. The proposed model was then applied to ESR prediction for patients with a particular disease. The aim of this study can be summarized as:

- To reduce measurement time of ESR, using ensemble deep learning models.
- To find the best performing ensemble model based on the statistical scores.
- To validate the applicability of the selected ensemble model for predicting ESR in nontrained samples (Patients with a particular disease).

## Material and methods

### Blood sample preparation

A total of 310 subjects from the Pusan National University Dental Hospital (Busan, Korea) participated in this study. The study population included 304 normal subjects and 6 patients who have periodontal disease. The age of the subjects ranged from 5 to 95 years (48.369 ± 18.823). The percentages of males and females were 46% and 54%, respectively. Blood samples were collected via 3-mL BD Vacutainer® K2 EDTA tubes (Becton, Dickinson and Co., Franklin Lakes, NJ) for anticoagulation. A centrifuge (DSC-102SD, Nasco Korea) was used for separating erythrocytes from plasma. Separated erythrocytes were mixed with autologous plasma with the hematocrit (packed volume percentage of erythrocytes) fixed at 35% by mixing. Ethical permission for this study was granted by the institutional review board (IRB) of Pusan National University Hospital (H- 1911–028–085), and the study followed the rules of the Declaration of Helsinki.

### Experimental setup

**Figure 1:**
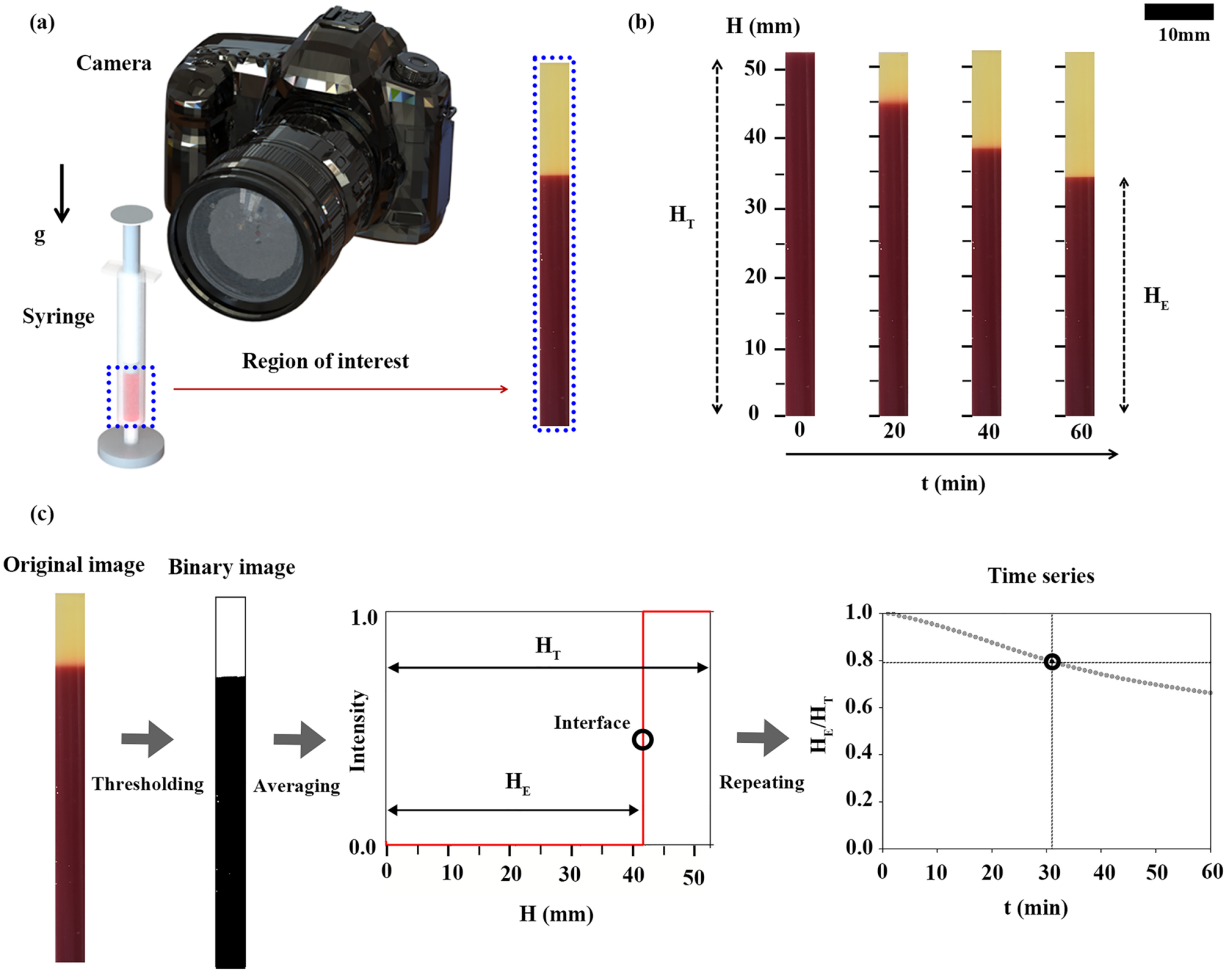
(**a**) Measurement system composed of a disposable syringe (1 mL) and digital camera. (**b**) Typical images of sedimented erythrocytes in a blood sample with respect to time (t). Height of the sedimented erythrocytes (H_E_) is gradually decreased during 60 min. (**c**) Data processing procedure. The original image is transformed into a binary image using Otsu’s method. The binary image is averaged in the horizontal direction to detect an interface (marked with a black circle) between sedimented erythrocytes and plasma. This interface, denoted by H_E_/H_T_, was extracted during 60 min (marked with a gray circle), and forms time series data.

Figure 1a shows the experimental setup for recording the images for erythrocyte sedimentation. A disposable syringe (1 mL syringe, BD, USA) filled with a 1 mL blood sample was mounted vertically. Consecutive images of the blood in the syringe were acquired by a digital camera (D5600, Nikon Imaging Korea Co., Ltd., Korea) with a micro-lens (AF Micro-Nikkor 105 mm f/2.8D, Nikon Co., Japan). Using software (Camera control pro 2, Nikon Co., Japan), images were taken automatically at intervals of 0.5 min for 60 min. The pixel size of the CMOS camera was 6000 × 4000 pixels. All experiments were conducted at room temperature (25 °C) with 50% humidity.

### Image processing procedure

Figure 1b shows the process of erythrocyte sedimentation in a blood sample at various times (t = 0, 20, 40, and 60 min). The total height of the blood (H_T_) was about 53 mm, corresponding to 1 mL. The height of the sedimented erythrocytes (H_E_) decreased continuously due to gravity sedimentation with the lapse of time. Figure 1c shows the image processing procedure using commercial software (MATLAB, Mathworks, USA). The captured images were cropped to the region of interest in the syringe (120 × 2900 pixels) and then converted into gray images. The images were transformed into binary images using Otsu’s method. By averaging the binary images in the horizontal direction, variation of the intensity along the gravity direction was obtained. The interface between sedimented erythrocytes and separated plasma was detected by utilizing the ‘findchangepts’ function in MATLAB, which detects a drastic change in signal. Based on the detected interface, H_E_/H_T_ was determined at a given time. During ESR measurement (60 min), temporal variation of H_E_/H_T_ for one sample was obtained by repeating the procedure, as illustrated in the right inset of Fig. 1c. For learning and training for the prediction of ESR, normalized time series data (H_E_/H_T_) were used as model input.

### Deep learning methodology for ESR prediction

#### MLP model

The MLP is the most widely used algorithm for forecasting. It consists of multiple layers of interconnected elements called neurons with a nonlinear activation function (φ)^35^. Each neuron is only forward connected to every neuron in the subsequent layers. This structure is combined with the hidden layer activation function and allows the MLP to approximate any continuous function^36^. The mathematical model of the MLP is represented by:

1 $${\mathrm{y}}_{\mathrm{i}}=\mathrm{\varphi }\left(\sum_{\mathrm{i}=1}^{\mathrm{H}}{\mathrm{w}}_{\mathrm{ij}}{\mathrm{x}}_{\mathrm{i}}+{\mathrm{b}}_{\mathrm{j}}\right)$$

where y_i_ is the output variable, H represents the number of neurons in the hidden layer, w_ij_ is the weight connecting the jth neuron and the ith neuron, x_i_ is an input variable, and b_j_ is the bias of the jth neuron.

#### LSTM

A recurrent neural network (RNN) has a recurrent hidden state with an activation at each time that depends on the one from the previous time. RNN based algorithms have been developed in time series forecasting applications^37–39^. Due to infinite lookback windows, RNNs are vulnerable to long-range dependencies in the data, and the gradients tend to either vanish or explode^40^. LSTM is a variant of RNN that was developed to deal with the limitation of RNNs by using memory cells. A memory cell in each LSTM unit stores long-term information and these cells are modulated through three gates: an input gate (i_t_), output gate (o_t_), and forget gate (f_t_). These gates adjust the hidden state (h_t_) and cell states (c_t_) of the LSTM as follows:

2 $${\mathrm{i}}_{\mathrm{t}}=\upsigma \left({\mathrm{W}}_{\mathrm{i}1}{\mathrm{X}}_{\mathrm{t}}+{\mathrm{W}}_{\mathrm{i}2}{\mathrm{h}}_{\mathrm{t}-1}+{\mathrm{b}}_{\mathrm{i}}\right)$$

3 $${\mathrm{o}}_{\mathrm{t}}=\upsigma \left({\mathrm{W}}_{\mathrm{o}1}{\mathrm{X}}_{\mathrm{t}}+{\mathrm{W}}_{\mathrm{o}2}{\mathrm{h}}_{\mathrm{t}-1}+{\mathrm{b}}_{\mathrm{o}}\right)$$

4 $${\mathrm{f}}_{\mathrm{t}}=\upsigma \left({\mathrm{W}}_{\mathrm{f}1}{\mathrm{X}}_{\mathrm{t}}+{\mathrm{W}}_{\mathrm{f}2}{\mathrm{h}}_{\mathrm{t}-1}+{\mathrm{b}}_{\mathrm{f}}\right)$$

5 $${{\mathrm{c}}_{\mathrm{t}}=\mathrm{f}}_{\mathrm{t}}\odot {\mathrm{c}}_{\mathrm{t}-1}+{\mathrm{i}}_{\mathrm{t}}\odot \mathrm{tanh}\left({\mathrm{W}}_{\mathrm{c}1}{\mathrm{X}}_{\mathrm{t}}+{\mathrm{W}}_{\mathrm{c}2}{\mathrm{h}}_{\mathrm{t}-1}+{\mathrm{b}}_{\mathrm{c}}\right)$$

6 $${\mathrm{h}}_{\mathrm{t}}={\mathrm{o}}_{\mathrm{t}}\odot \mathrm{tanh}({\mathrm{c}}_{\mathrm{t}})$$

where W and b are the weights and biases of the LSTM layer, respectively. σ is a sigmoid function, and tanh is a hyperbolic tangent function. ⊙ is the element-wise (Hadamard) product.

#### GRU

GRU was introduced by Cho et al.^16^ and makes each recurrent unit with an adaptive selection of dependencies of different time scales. Unlike LSTM, GRU modulates the flow of information inside the unit without memory cells. GRU has two gates: an update gate (g_t_) and reset gate (r_t_).

7 $${\mathrm{g}}_{\mathrm{t}}=\upsigma \left({\mathrm{W}}_{\mathrm{z}1}{\mathrm{X}}_{\mathrm{t}}+{\mathrm{W}}_{\mathrm{z}2}{\mathrm{h}}_{\mathrm{t}-1}+{\mathrm{b}}_{\mathrm{z}}\right)$$

8 $${\mathrm{r}}_{\mathrm{t}}=\upsigma \left({\mathrm{W}}_{\mathrm{r}1}{\mathrm{X}}_{\mathrm{t}}+{\mathrm{W}}_{\mathrm{r}2}{\mathrm{h}}_{\mathrm{t}-1}+{\mathrm{b}}_{\mathrm{r}}\right)$$

9 $${\widetilde{\mathrm{h}}}_{\mathrm{t}}=\mathrm{tanh}\left({\mathrm{W}}_{\mathrm{h}1}{\mathrm{X}}_{\mathrm{t}}+{\mathrm{W}}_{\mathrm{h}2}\left({\mathrm{r}}_{\mathrm{t}}\odot {\mathrm{h}}_{\mathrm{t}-1}\right)+\mathrm{b}\right)$$

10 $${\mathrm{h}}_{\mathrm{t}}=\left(1-{\mathrm{g}}_{\mathrm{t}}\right)\odot {\mathrm{h}}_{\mathrm{t}-1}+{\mathrm{g}}_{\mathrm{t}}\odot {\widetilde{\mathrm{h}}}_{\mathrm{t}}$$

where ${\widetilde{\mathrm{h}}}_{\mathrm{t}}$ denotes candidate hidden state.

### Selection of sequence length

To reduce ESR measurement time, the length of the input sequence of the prediction should be decreased. The starting time is fixed as 0 min. To select the optimal length of time, different time spans were tested for MLP, LSTM, and GRU. The input time ranged from 5 to 20 min with intervals of 2.5 min. When the input time is shorter than 5 min, the predicted results are extremely poor.

### Ensemble model

**Figure 2:**
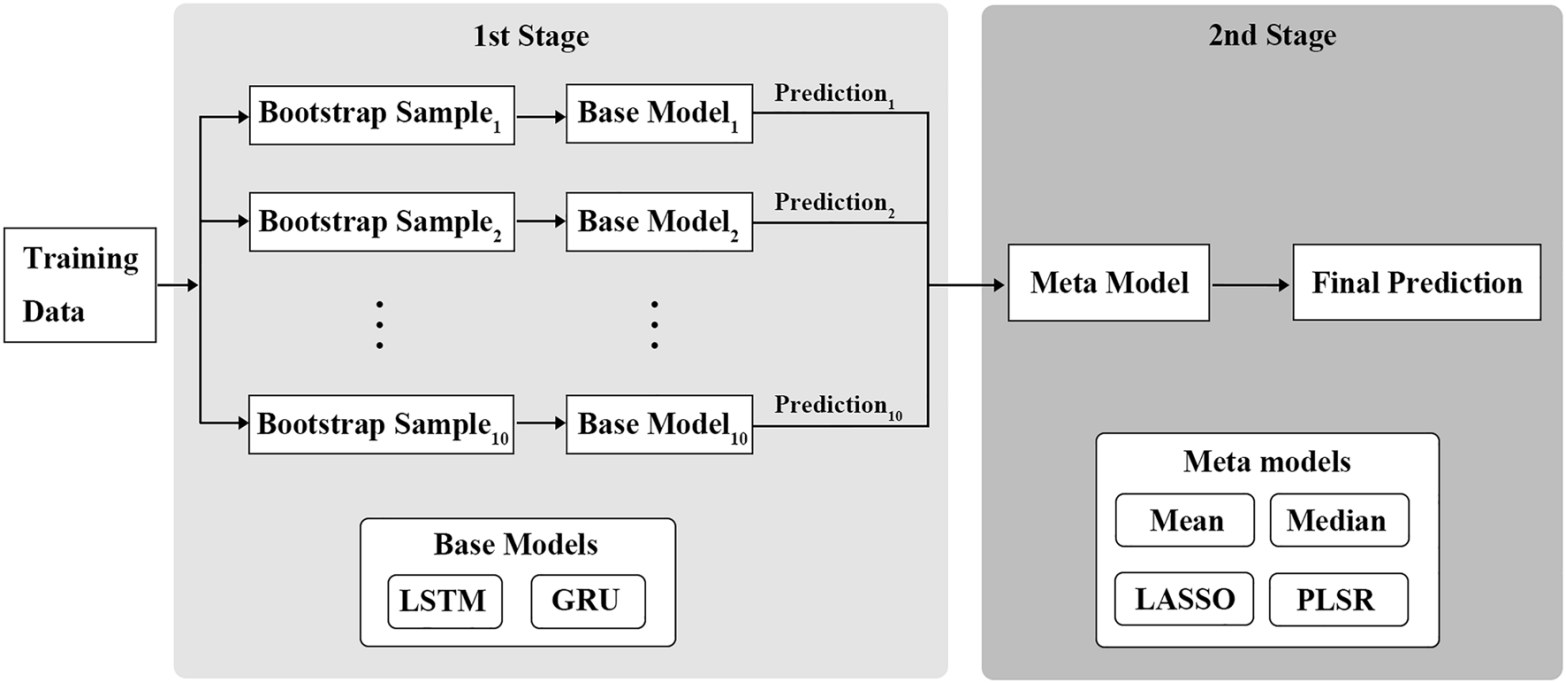
Structure of the ensemble model.

In the present work, stacking ensemble using bootstrapped samples of the training data was proposed. As shown in Fig. 2, stacking is a two-stage approach. In the first stage, multiple base models were built on bootstrap samples, and corresponding predictions were obtained. Bootstrap samples were randomly drawn with replacement from the entire training data. In the second stage, meta model combines the predictions of the base models and produces the final prediction. The final prediction should be a weighted average of the individual predictions since some base models are more accurate than others. However, given that all base models attempt to solve the same task, their predictions are expected to be highly correlated, which can lead to performance degradation of the ensemble^24,28,41^. Feature selection technique is necessary to remove the redundant or irrelevant features or features correlated in the data. Some studies proposed deep autoencoder or deep belief network to extract the nonlinear features^42,43^. However, the predictions of the base models didn’t show any complex patterns but high correlation in this study. Thus, LASSO and PLSR were used to cope with the multicollinearity due to the high degree of correlation between the individual predictions. The meta models are briefly introduced.

#### Mean ensemble

The mean is the simplest weight combination approach and assigns equal weights to all the individual forecasts^44^. The mean operator is intuitive, but it has a limitation that is widely known: the mean is susceptible to outliers and skewed distribution. The mean ensemble forecast (z_a_) can be calculated as:

11 $${\mathrm{z}}_{\mathrm{a}}=\frac{1}{\mathrm{M}}\sum_{\mathrm{i}=1}^{\mathrm{M}}{\mathrm{z}}_{\mathrm{i}}$$

where M is the number of base models, and z_i_ (i = 1, …, M) denotes the forecasted output vector of the ith base model.

#### Median ensemble

The median simply provides the middle value of z_i_ in a sorted list if M is odd or the mean of the two middle values otherwise^44^. The median is less affected by the influence of outliers. The median ensemble forecast (z_m_) can be calculated as:

12 $${\mathrm{z}}_{\mathrm{m}}=\left\{\begin{array}{c}{\mathrm{z}}_{(\mathrm{M}+1)/2},\quad\quad\quad\quad\quad if\, M\, is\, odd\\ {(\mathrm{z}}_{\mathrm{M}/2}+{\mathrm{z}}_{\mathrm{M}/2+1})/2,\quad if\, M\, is\, even\end{array}\right.$$

#### Least absolute shrinkage and selection operator (LASSO)

The LASSO is a regularized regression to deal with multicollinearity in data^45^. The LASSO penalty can force certain coefficients to be zero by imposing a penalty on their size, and it yields interpretable results with improved accuracy. The LASSO estimator (${\widehat{\upbeta }}_{\mathrm{L}})$ is the solution to the following optimization problem:

13 $${\widehat{\upbeta }}_{\mathrm{L}}=\underset{\upbeta }{\mathrm{arg min}}{\Vert \mathrm{y}-\mathrm{Z\upbeta }\Vert }_{2}^{2}+\uplambda {\Vert \upbeta \Vert }_{1}$$

where y represents the vector of observed ESR values, Z = [z_1_, z_2_, …, z_M_] is the individual forecasts matrix, and β denotes the regression coefficient. λ is a positive regularization (penalty/tuning) parameter that controls the amount of shrinkage of β. As the value of λ depends on the data, it can be determined using data-driven methods such as cross-validation (CV). The intercept is ignored for convenience. The LASSO ensemble forecast (z_L_) can be calculated as:

14 $${\mathrm{z}}_{\mathrm{L}}=\mathrm{Z}{\widehat{\upbeta }}_{\mathrm{L}}$$

#### Partial least squares regression (PLSR)

The basic idea of PLSR is to project the original variables onto a set of orthogonal latent variables. The latent variables, called components, are linear combinations of the original variables. The number of the latent components (J) is determined by CV, similar to the LASSO method. PLSR extracts these components by modeling both input and output data^46^. PLSR builds a relationship between the input matrix Z and the output vector y. The PLSR technique attempts to find a linear decomposition of Z and y by the outer relations^47^:

15 $$\mathrm{Z}={\mathrm{TP}}^{\mathrm{T}}+{\mathrm{E}}_{\mathrm{x}}=\sum_{\mathrm{j}=1}^{\mathrm{J}}{\mathrm{t}}_{\mathrm{j}}{\mathrm{p}}_{\mathrm{j}}^{\mathrm{T}}+{\mathrm{E}}_{\mathrm{x}}$$

16 $$\mathrm{y}={\mathrm{UQ}}^{\mathrm{T}}+{\mathrm{E}}_{\mathrm{y}}=\sum_{\mathrm{j}=1}^{\mathrm{J}}{\mathrm{u}}_{\mathrm{j}}{\mathrm{q}}_{\mathrm{j}}^{\mathrm{T}}+{\mathrm{E}}_{\mathrm{y}}$$

where T and U are score matrices, P and Q are loading matrices, and E_x_ and E_y_ are residual errors for input and output. p_j_ and q_j_ are loading vectors that can be considered as weights. The superscript “T” means transposition. Latent vectors t_j_ and u_j_ are obtained to maximize the covariance between Z and y while minimizing E_x_ and E_y_. The inner relation between U and T is given by:

17 $$\mathrm{U}=\mathrm{TB}+{\mathrm{E}}_{\mathrm{U}}$$

where B denotes the coefficients, and E_U_ is an error term between U and T. If the error terms are minimized, the PLSR ensemble forecast (z_P_) can be calculated as:

18 $${\mathrm{z}}_{\mathrm{P}}=\sum_{\mathrm{j}=1}^{\mathrm{J}}{\mathrm{u}}_{\mathrm{j}}{\mathrm{q}}_{\mathrm{j}}^{\mathrm{T}}$$

### Deep neural network implementation

DNN models ware built using TensorFlow 2.8.0 and Keras packages in Python 3.7.12 in the Google Colaboratory environment. For training and evaluation of the model, time series data of 304 normal subjects were randomly split into training, validation, and test sets (194, 50 and 60 subjects, respectively). Models were trained for 300 epochs with an early stopping method. Early stopping was adopted that specifies an arbitrarily large number of epochs and stops training the model when there is no improvement in the validation loss for 15 epochs. Moreover, an exponential decay schedule was utilized, which decays the learning rate using an exponential function. The loss function was the mean squared error. The optimizer used in this study was the adaptive moment estimation (ADAM), which showed the highest performance among other optimizers including Adadelta, Adagrad, RMSprop, and SGD in a preliminary test.

The neural network architecture was determined empirically by monitoring the performance with different architectures. MLP consisted of two hidden layers: one with 15 neurons and one with 10 neurons. The architecture of the GRU consisted of one hidden layer with 15 neurons. The architecture of LSTM was identical to that of GRU. A dense layer with a single neuron was chosen for the output layer in all DNNs.

### Performance metrics

The forecasting performance of each model was evaluated through some error metrics. The mean absolute percentage error (MAPE) and root mean squared error (RMSE) were adopted^48^. The formulas for MAPE and RMSE are as follows:

19 $$\mathrm{MAPE}=\frac{1}{{\mathrm{n}}_{\mathrm{s}}}\sum_{\mathrm{i}=1}^{{\mathrm{n}}_{\mathrm{s}}}\left|\frac{{\mathrm{y}}_{\mathrm{i}}-{\widehat{\mathrm{y}}}_{\mathrm{i}}}{{\mathrm{y}}_{\mathrm{i}}}\right|\times 100 \left(\mathrm{\%}\right)$$

20 $$\mathrm{RMSE}=\sqrt{\frac{1}{{\mathrm{n}}_{\mathrm{s}}}\sum_{\mathrm{i}=1}^{{\mathrm{n}}_{\mathrm{s}}}{\left({\mathrm{y}}_{\mathrm{i}}-{\widehat{\mathrm{y}}}_{\mathrm{i}}\right)}^{2}}$$

where n_s_ and ŷ denote the number of samples and the forecasted ESR, respectively.

### Statistical test for all pairwise comparison

The Friedman test is a non-parametric test that detects significant differences among models^49^. It produces ranks of models’ performance averaged over multiple datasets. The null hypothesis of the Friedman test states that all the models are equivalent and their mean ranks are nearly equal. If the null hypothesis is rejected with very small p-values, the Nemenyi post-hoc test can be done^50^. Although the Nemenyi test is known to be conservative, the procedure is the simplest among post-hoc tests for pairwise multiple comparisons^51^. The Nemenyi test was used to compare all models with each other. The performance between any two models is significantly different if their corresponding mean ranks differ by at least the critical distance (CD). The CD is given by:

21 $$\mathrm{CD}={\mathrm{q}}_{\mathrm{\alpha }}\sqrt{\frac{\mathrm{k}\left(\mathrm{k}+1\right)}{6\mathrm{D}}}$$

where k and D are the numbers of models and datasets, respectively, and ${\mathrm{q}}_{\mathrm{\alpha }}$ is the critical value based on studentized range statistics divided by $\sqrt{2}$.

## Results and discussion

### Tendency of the erythrocyte sedimentation

**Figure 3:**
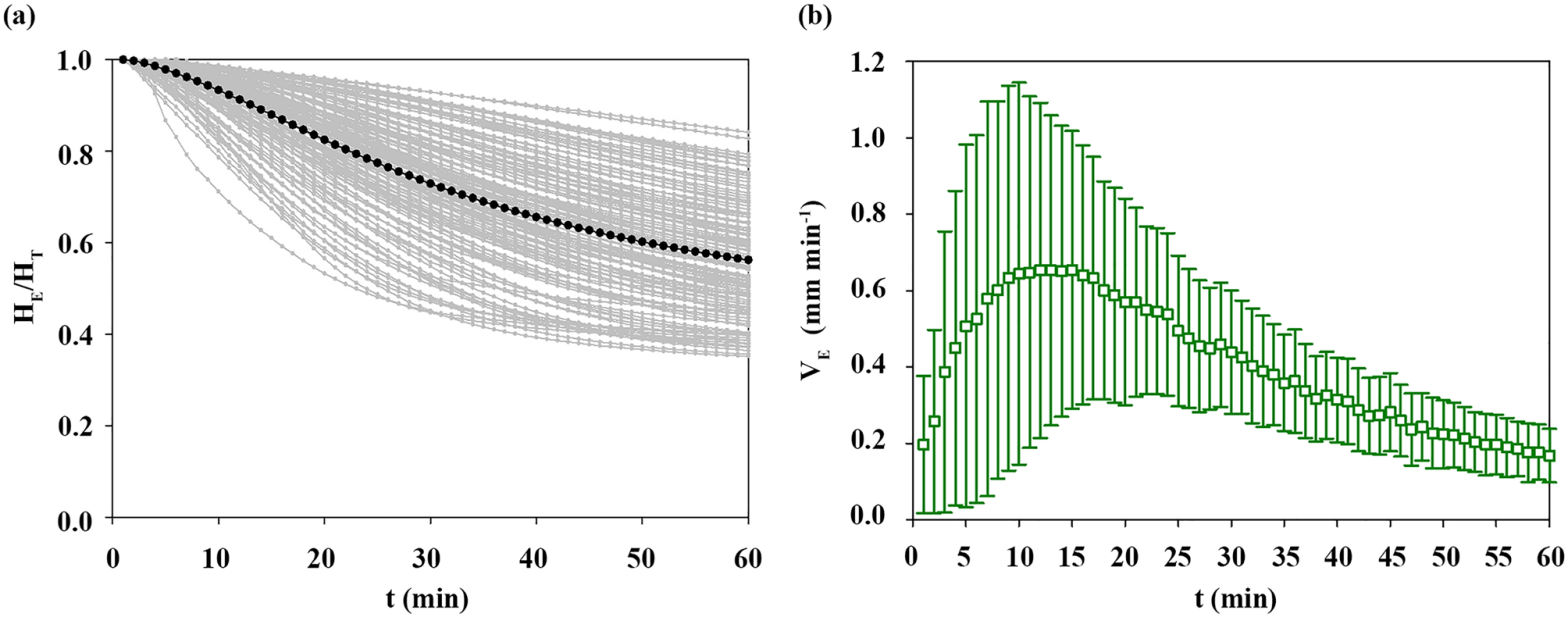
(**a**) Temporal variations of H_E_/H_T_ for 304 normal subjects. Mean value of every H_E_/H_T_ is marked by black dotted line. (**b**) Instantaneous time rate of sedimentation (V_E_). Velocity data are represented as mean ± SD (n = 304).

Figure 3a shows the temporal variations of H_E_/H_T_ for all subjects (n = 304) and mean trend of all samples (black dotted line). Since the hematocrit of blood samples was fixed at 35%, ESR values (H_E_/H_T_ at 60 min) ranged from 0.350 to 0.859. Although some samples show similar ESR values, the variation tendency of H_E_/H_T_ is somewhat different. To further investigate the variation tendency during the erythrocyte sedimentation process, the instantaneous time rate of sedimentation (V_E_ = −dH_E_/dt) for 304 subjects is shown in Fig. 3b as the mean and standard deviation (SD). The extent of V_E_ can be approximately divided into two stages according to time. Within 13 min, V_E_ shows an acceleration stage. After that, it tends to decrease with the lapse of time. Since V_E_ can describe the extent of erythrocyte sedimentation for a specific time, it is closely related to the ESR sedimentation process.

The sedimentation process can be approximately described by 3 phases: aggregation, sedimentation, and packing^52,53^. During the aggregation phase, erythrocytes aggregate and form the rouleaux or aggregates. Since the sedimentation speed is proportional to the particle size^6^, large rouleaux and aggregates produced in the aggregation stage promote sedimentation. As a result, erythrocytes rapidly fall in the sedimentation phase, whereas erythrocytes slowly fall and pile up at the bottom of the tube in the packing phase. It can be inferred that times with high V_E_ (10–20 min) may be included in the sedimentation phase (Fig. 3b). Thus, temporal variations of H_E_/H_T_ at 0–20 min are important to predict the ESR value because they provide information on the erythrocyte sedimentation process.

### Performance comparison of DNNs depending on input sequence lengths

**Figure 4:**
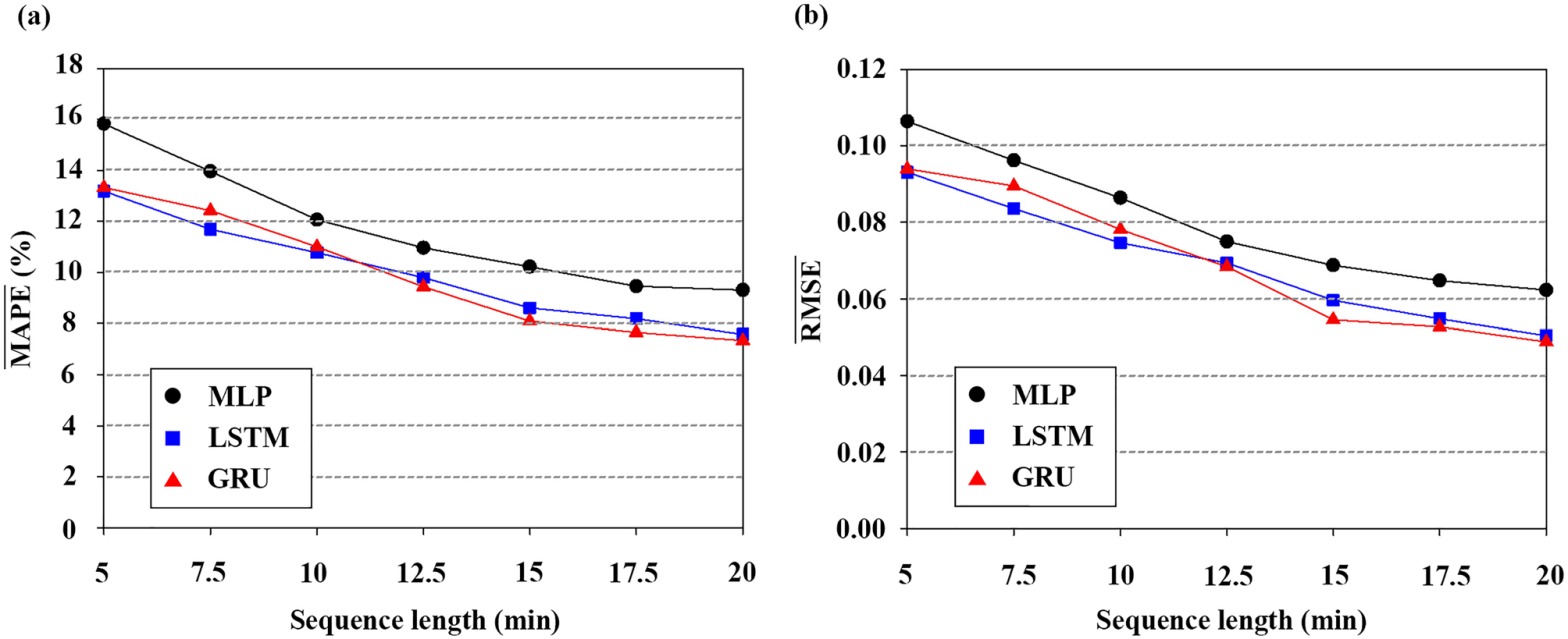
(**a**) $\overline{\mathrm{MAPE} }$ (%) of the individual models according to sequence length (min). $\overline{\mathrm{MAPE} }$ is the mean MAPE over 30 repeated experiments. (**b**) $\overline{\mathrm{RMSE} }$ of the individual models according to sequence length (min). $\overline{\mathrm{RMSE} }$ is the mean RMSE over 30 repeated experiments.

Figure 4a,b show the variations of $\overline{\mathrm{MAPE} }$ and $\overline{\mathrm{RMSE} }$ according to the sequence length (input data at 0-t min). $\overline{\mathrm{MAPE} }$ and $\overline{\mathrm{RMSE} }$ are mean values of MAPE and RMSE obtained by repeating the prediction over 30 times. Due to the random setting of initial weights and biases, the 30 results are different. In general, prediction errors decrease with increasing sequence length for all DNNs. MLP shows relatively poor performance for all sequence lengths. This might result from the inherent structure of MLP, which cannot learn the sequential order of the time series input. As shown in Supplementary Table 1, SD values of MLP are usually higher than those of LSTM and GRU. This indicates that the prediction results of LSTM and GRU are less dependent on the initial setting than those of MLP. These results are well matched with a previous study that compared the prediction uncertainty of LSTM, GRU, and ANN^54^. Since similar accuracy was observed between LSTM and GRU, the optimal sequence length was determined based on LSTM and GRU results. $\overline{\mathrm{MAPE} }$ and $\overline{\mathrm{RMSE} }$ of both GRU and LSTM gradually decrease until the sequence length reaches 15 min and show a marginal decrease from 15 min onward.

Determination of the exact time input for the ESR is challenging since many factors also affect ESR, including sex, age, and even vertical installation angle of the specimen tubes. Previous studies reported that the ESR can be estimated by the ESR recorded at 30 min^9,55^. The inconsistent optimal time can be explained by the method to monitor erythrocyte sedimentation. In this experiment, relatively dense temporal variations of erythrocyte sedimentation (time interval of 0.5 min) were recorded. In contrast, previous reports measured the ESR values at 15, 20, 30, and 40 min. Considering the marginal decrease from 15 min with small values of $\overline{\mathrm{MAPE} }$ and $\overline{\mathrm{RMSE} }$, the optimal sequence length was selected as 15 min.

### Performance of ensemble model according to ensemble size

As the number of base models (N_B_) is an important parameter in ensemble performance, its impact was investigated in terms of $\overline{\mathrm{MAPE} }$ and $\overline{\mathrm{RMSE} }$. Both LSTM and GRU were selected as base models for the ensemble. All meta models were implemented in MATLAB, where the parameters of LASSO and PLSR were determined by fivefold CV. All base models were trained on bootstrap samples and run 30 times for the fixed sequence length (15 min) of the test set. To select an adequate ensemble size, N_B_ was varied from 3 to 10. In a preliminary test, $\overline{\mathrm{MAPE} }$ and $\overline{\mathrm{RMSE} }$ of each ensemble model were almost saturated when N_B_ exceeded 10 DNNs.

**Figure 5:**
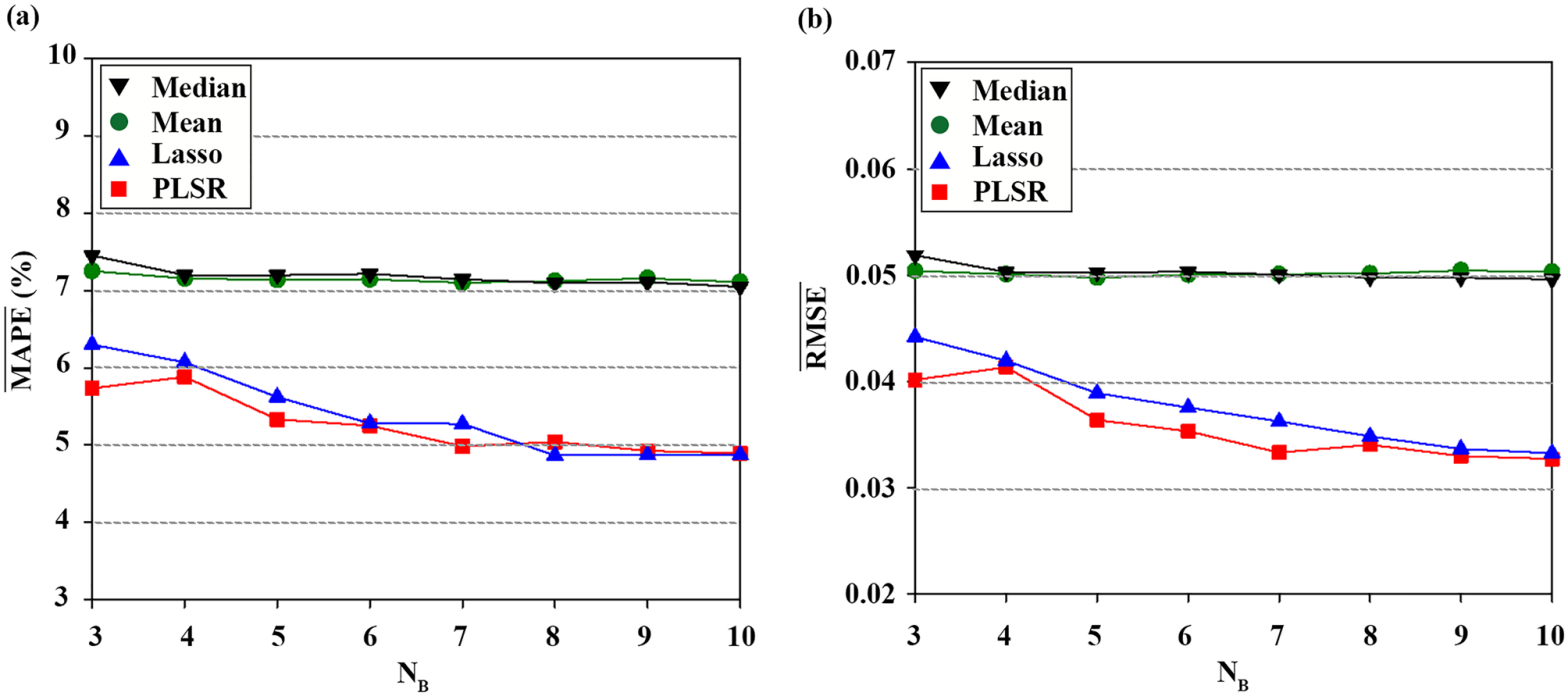
Forecasting accuracy of LSTM-based ensemble model according to number of base models (N_B_): (**a**) $\overline{\mathrm{MAPE} }$ (%) for different meta models, (**b**) $\overline{\mathrm{RMSE} }$ for different meta models.

**Figure 6:**
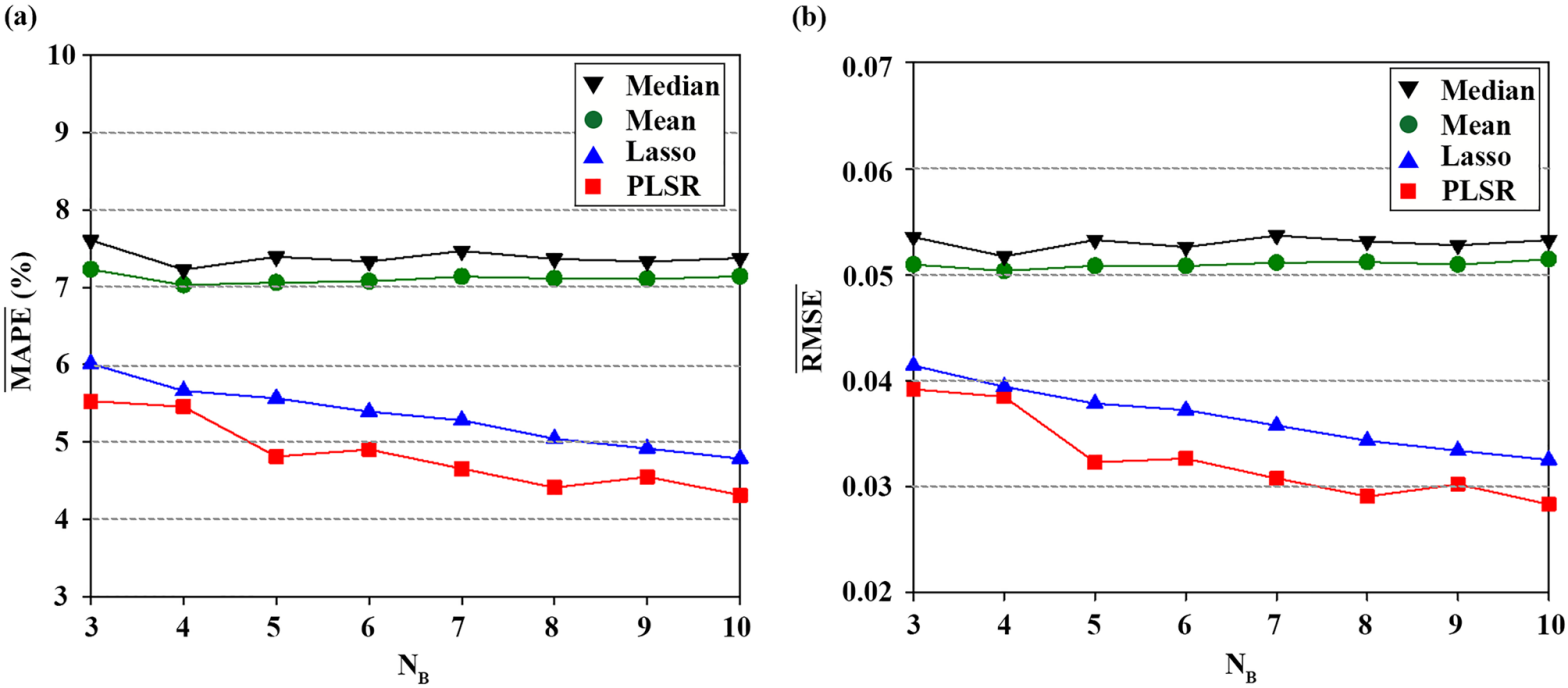
Forecasting accuracy of GRU-based ensemble model according to N_B_: (**a**) $\overline{\mathrm{MAPE} }$ (%) for different meta models, (**b**) $\overline{\mathrm{RMSE} }$ for different meta models.

Figures 5,6 show the variations of $\overline{\mathrm{MAPE} }$ and $\overline{\mathrm{RMSE} }$ for LSTM and GRU coupled with four meta models. It can clearly be observed that the results of the mean are similar to those of the median. This marginal difference can be explained by the strong correlation between the individual forecasts. As the mean and median are measures of the central tendency, multicollinearity in the individual forecasts results in similar performance between them. However, LASSO and PLSR methods are more accurate than the mean and median over all N_B_, which show superior ability to handle the multicollinearity.

Combinations of LSTM with PLSR and LASSO show similar performance, as illustrated in Fig. 5. However, compared to LASSO, PLSR shows a different trend for N_B_ ranging from 3 to 7. Particularly, PLSR exhibits a significantly low error when N_B_ increases from 4 to 5. Both methods show marginal improvement with the increase in N_B_ (N_B_ > 8). In the case of the GRU base model, PLSR achieves better results, followed by LASSO (Fig. 6). The variations of PLSR fluctuate with increasing N_B_, whereas the variations of LASSO gradually decrease. PLSR shows relatively high reduction of error when N_B_ varies from 4 to 5, which was similarly observed in LSTM results (Fig. 5).

Although PLSR shows the minimum error at N_B_ of 10, there is no significant difference at N_B_ ranging from 8 to 10 in terms of PLSR performance (Figs. 5,6). The performance degradation with the increase in N_B_ indicates that usage of all base models may not contribute to the improvement of the ensemble forecast^56,57^. The pattern learned by some base models has already been represented by other models due to serious correlation between the models when N_B_ is sufficiently high. Thus, the candidates for the optimal N_B_ are 8 and 10. In this study, the optimal N_B_ was determined as 8 because there was no significant difference between 8 and 10.

### Statistical comparison of model performance

**Figure 7:**
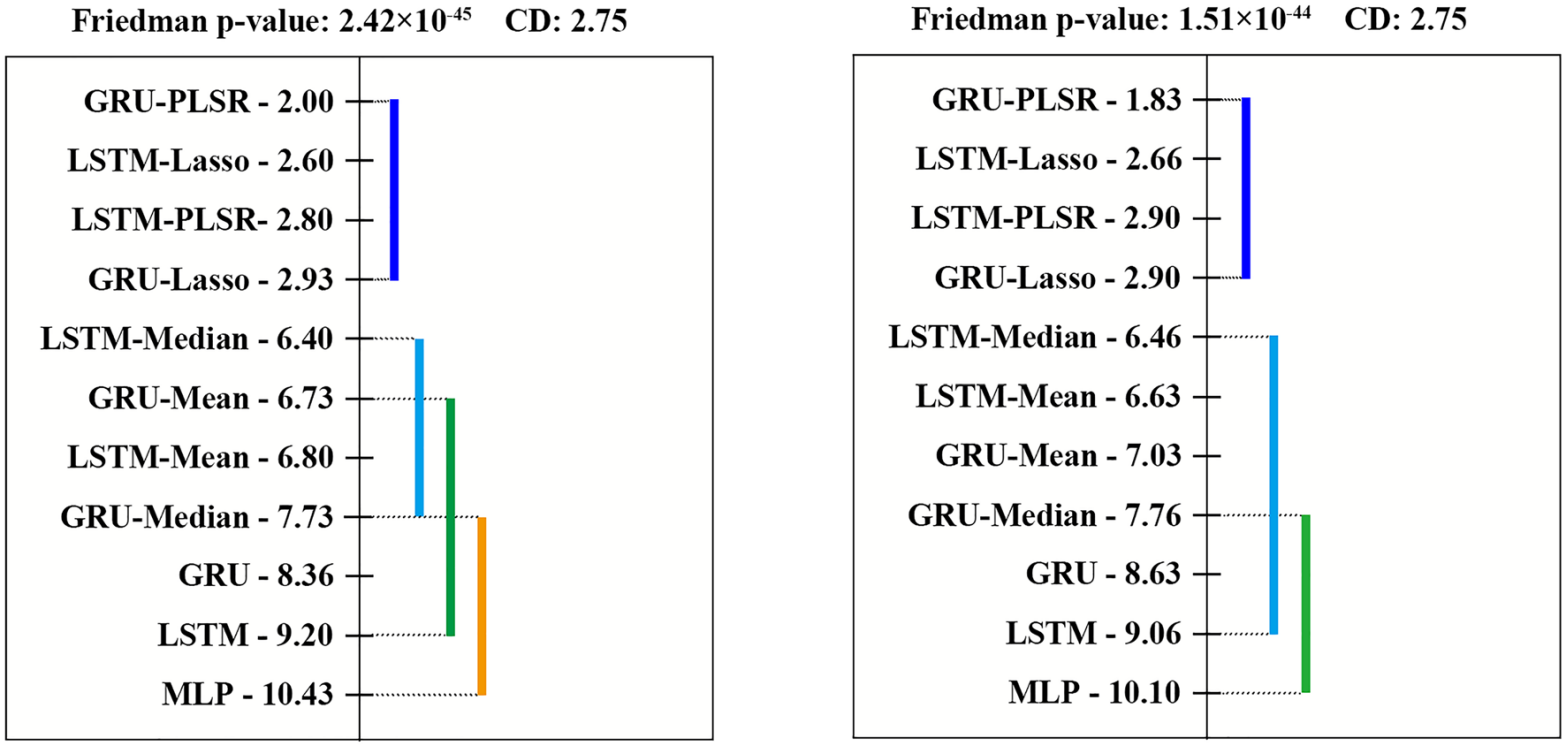
Nemenyi test results based on MAPE (left) and RMSE (right) for N_B_ of 8. The critical distance (CD) is 2.75.

To examine significant differences among all models, statistical tests were carried out with the selected N_B_ of 8. Extremely low p-values were obtained by the Friedman test for both MAPE and RMSE at a significance level of 0.05, so the Nemenyi test was performed as illustrated in Fig. 7. P-values and CD are shown in the title, and the mean rank is marked next to each model. The ensemble models are named based on their base models and the meta models, such as LSTM-Mean or GRU-Median. As the base models coupled with meta models show better performance (low mean ranks), the models are positioned at the top in ascending order. The methods are not significantly different if their mean ranks share the same color bar that is less than or equal to the CD.

According to the Nemenyi test rank, GRU–PLSR achieves the lowest mean rank in both of $\overline{\mathrm{MAPE} }$ and $\overline{\mathrm{RMSE} }$. Furthermore, the ensemble models coupled with PLSR and LASSO outperform the other meta models and the individual models with 95% confidence. The findings suggest that both PLSR and LASSO are useful meta models and that GRU–PLSR is the best-performing ensemble model for the ESR prediction.

### Application of GRU–PLSR for ESR prediction of periodontitis patients

**Figure 8:**
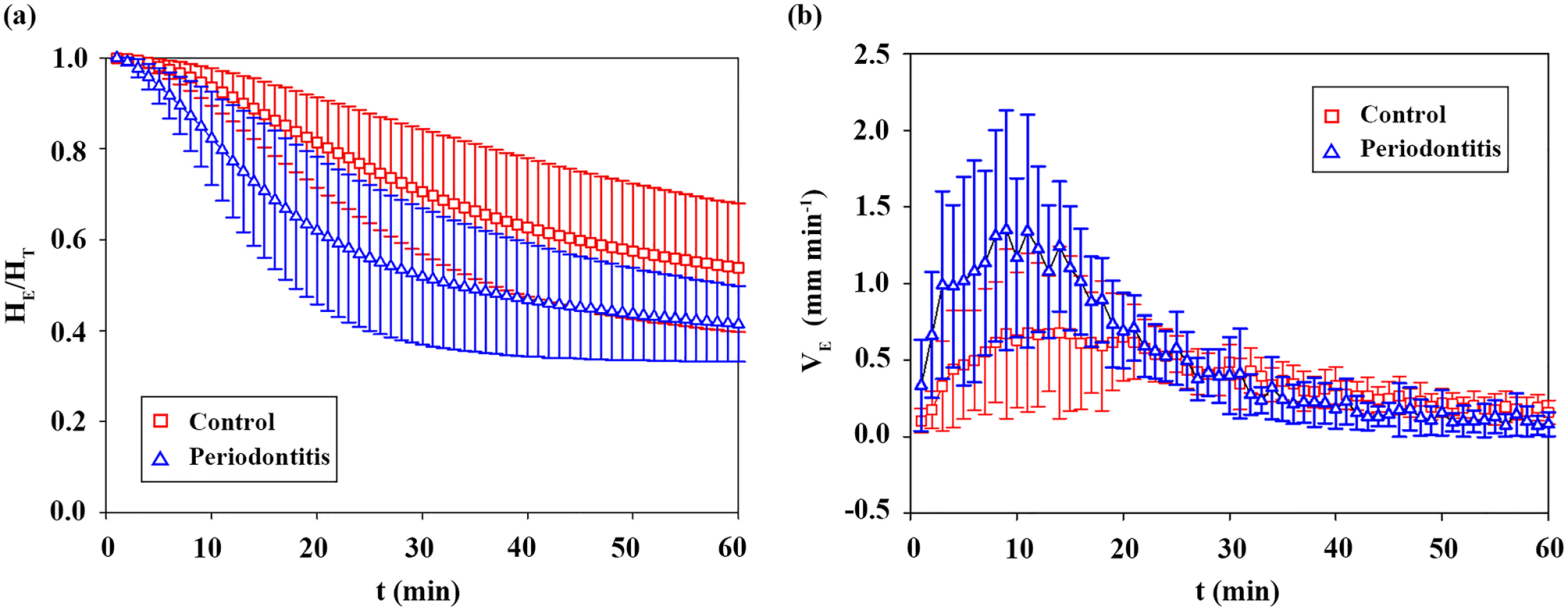
(**a**) Temporal variation of H_E_/H_T_ for the control and periodontitis subjects. (**b**) Instantaneous time rate of sedimentation of V_E_ for the normal subjects and periodontitis patients. All data shown are values of mean ± SD (n = 6).

Periodontal disease is a bacterial infection that damages the supporting tissues of the teeth. It may result in tooth loss and elevation of the body’s inflammation. Some studies reported relatively high ESR values in patients with periodontitis disease^58,59^. The ESR difference between normal subjects and periodontitis patients was monitored to examine the effect of periodontitis disease. For comparison, 6 control subjects were randomly sampled from 304 normal subjects. Temporal variation of H_E_/H_T_ for the control group and periodontitis patients is illustrated in Fig. 8a.

The ESR tendency shows differences between the two groups because ESR is increased by infectious and inflammatory diseases. The mean values of ESR for the control and periodontitis groups are 0.537 ± 0.141 and 0.414 ± 0.083, respectively. This indicates that a fast sedimentation trend was observed in periodontitis patients. The different tendency from periodontitis disease is similar to a previous result of the faster sedimentation for a patient group in a comparison of characteristic time^8^. To further examine the effect of periodontitis disease, variations of V_E_ for the control and periodontitis groups are illustrated in Fig. 8b. Based on V_E_, the erythrocytes for periodontitis patients rapidly decreased in 15 min in comparison to the control subjects, which indicates that the periodontitis disease accelerates the aggregation and sedimentation phases.

**Figure 9:**
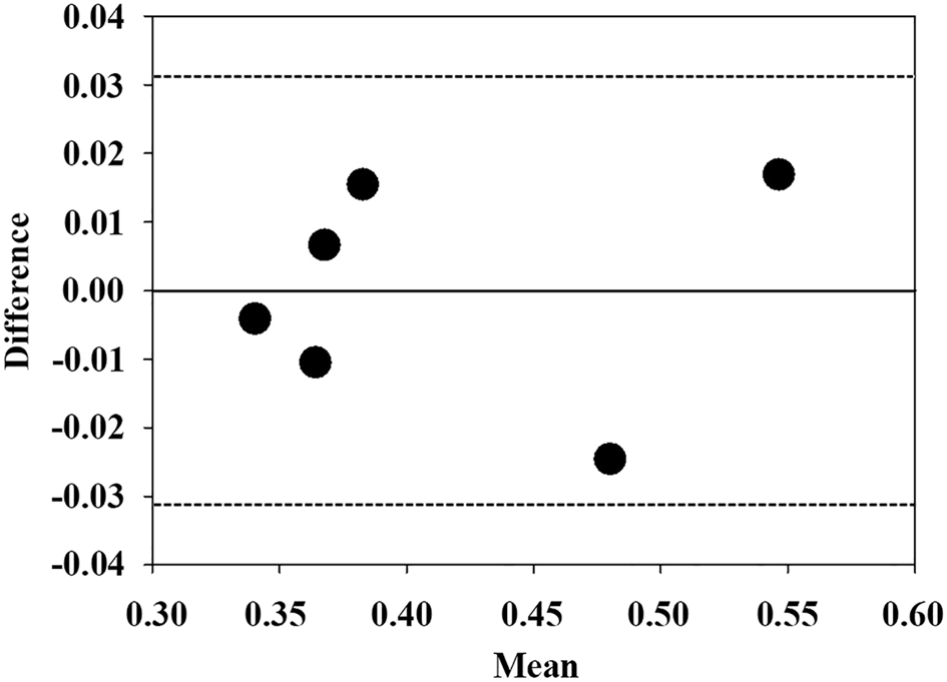
Bland–Altman plot demonstrating difference between the actual ESR values and predicted ESR values obtained by the GRU–PLSR model for periodontitis subjects. The horizontal axis represents the mean ESR values estimated by the two values, and the vertical axis shows the difference between them. The bold line and dashed lines denote the mean value of bias and 95% limits of agreement, respectively (n = 6).

GRU–PLSR trained using normal subjects was applied to the ESR prediction for periodontitis patients to demonstrate the practical applicability of the ensemble model. The Bland–Altman method was used to compare the similarity between methods^60^. Figure 9 displays the difference between the actual ESR values and predicted ESR values according to their mean values. The bold line and dashed lines denote the mean value of bias and 95% limits of agreement, respectively. As the presence of data in the 95% limits indicates that the two methods are well matched, ESR values estimated by the GRU–PLSR are in good agreement with the actual ESR values. However, the limited sample size of patients with periodontitis may lead to biased results, and further study with more data is needed.

### Contributions of the study

To the best of our knowledge, this is the first paper that analyzes and predicts the sedimentation tendency of erythrocytes using deep learning algorithms. The key contributions of this study are as follows:

- The use of deep learning approaches to predict ESR, in an attempt to find faster means of obtaining the ESR
- The use of ensemble models to improve the generalization ability on unseen data.
- The performance evaluation of various deep learning and ensemble models to find a best predictive model for ESR based on post-hoc analysis.

## Conclusion

In this study, the optimal sequence length was determined based on LSTM and GRU to reasonably predict the ESR value before 60 min. For improvement of ESR prediction, ensemble models with four meta models were suggested and compared. Empirical and statistical results demonstrated that PLSR and LASSO were superior meta models, and GRU–PLSR achieved the lowest mean ranks among all methods. Finally, the GRU–PLSR model was applied to the prediction of ESR values for patients with periodontitis. Based on our results, it can be concluded that GRU–PLSR is able to predict ESR values from inflammatory and non-inflammatory conditions with high accuracy in only 15 min. Therefore, GRU–PLSR could be useful for clinical applications in ESR measurement, where prompt decision-making is needed.

## Supplementary Information

Supplementary Information.

Supplementary Table 1.

## Data Availability

The datasets used and analysed during the current study available from the corresponding authors on reasonable request.
