# Supplementary material for: Neural network ensemble model for prediction of erythrocyte sedimentation rate (ESR) using partial least squares regression
Source: Sci Rep. 2022 Nov 15;12:19618. doi: 10.1038/s41598-022-23174-0 (PMC9666533; doi:10.1038/s41598-022-23174-0)
Supplement: Supplementary file 2 — Supplementary Table 1. [file 41598_2022_23174_MOESM2_ESM.docx]

**Supplementary Table 1.** The forecasting results by MLP, LSTM and GRU models with different sequence lengths. All data shown are values of the mean ± SD (n = 30).

| **Sequence lengths (min)** | **DNN model** | **MAPE (%)** | **RMSE** |
| --- | --- | --- | --- |
| 5 | MLP | 15.829 ± 2.017 | 0.106 ± 0.010 |
|  | LSTM | 13.192 ± 1.519 | 0.093 ± 0.011 |
|  | GRU | 13.335 ± 1.110 | 0.094 ± 0.009 |
|  |  |  |  |
| 7.5 | MLP | 13.961 ± 2.768 | 0.096 ± 0.017 |
|  | LSTM | 11.694 ± 1.298 | 0.083 ± 0.009 |
|  | GRU | 12.423 ± 1.738 | 0.089 ± 0.014 |
|  |  |  |  |
| 10 | MLP | 12.068 ± 1.621 | 0.086 ± 0.014 |
|  | LSTM | 10.776 ± 1.371 | 0.074 ± 0.008 |
|  | GRU | 11.006 ± 1.820 | 0.078 ± 0.012 |
|  |  |  |  |
| 12.5 | MLP | 10.966 ± 2.295 | 0.075 ± 0.012 |
|  | LSTM | 9.805 ± 1.562 | 0.069 ± 0.010 |
|  | GRU | 9.439 ± 1.264 | 0.068 ± 0.009 |
|  |  |  |  |
| 15 | MLP | 10.222 ± 2.411 | 0.068 ± 0.014 |
|  | LSTM | 8.613 ± 1.648 | 0.059 ± 0.010 |
|  | GRU | 8.097 ± 1.640 | 0.054 ± 0.011 |
|  |  |  |  |
| 17.5 | MLP | 9.461 ± 2.753 | 0.064 ± 0.017 |
|  | LSTM | 8.194 ± 1.981 | 0.054 ± 0.013 |
|  | GRU | 7.655 ± 1.245 | 0.052 ± 0.009 |
|  |  |  |  |
| 20 | MLP | 9.309 ± 2.317 | 0.062 ± 0.014 |
|  | LSTM | 7.578 ± 1.082 | 0.050 ± 0.008 |
|  | GRU | 7.336 ± 1.364 | 0.048 ± 0.009 |
